# Clinical Notes Taken in the Parisian Hospitals

**Published:** 1846-08

**Authors:** 


*Clinical notes taken in the Parisian Hospitals.—Asthma.*—According to M. Cruveilhier, the essential pathological condition of asthma does not consist in pulmonary emphysema, as is generally believed, but rather in spasm of the bronchial vesicles, which renders respiration incomplete. In fact, pulmonary emphysema is often wanting in asthmatics; it does not exist in a patient just now in M. Cruveilhier's ward, and yet the accessions at night are in her extremely grave; the respiration is sibilant, which, according to that Professor, is dependent on spasmodic contraction of the bronchial vesicles. One of the characters he has observed in asthmatics is a sense of compression in the chest from before backwards. He has more confidence in bleedings than in ammonia in combating this affection.

*Digitalis.*—M. Andral prefers administering digitalis by the rectum in the form of infusion. Hence he is obliged to prescribe large doses to obtain any effect. In a patient with diseased heart, he is giving this moment two grammes (40 grains) a day, infused in eight ounces of water as an injection. This mode of absorption, from its uncertainty, cannot be so valuable as that by the stomach.

*Variola.*—According to M. Chomel, the eruption of variola does not fade away in the order of its appearance. That on the arch of the palate, and on the other mucous membranes, vanishes first, then that on the scrotum, then that on the face, and, lastly, that on the trunk.

*Zona.*—According to M. Rayer, the acute inflammatory affection, which is called zona, is accompanied by two orders of phenomena; the one nervous, the other inflammatory,—the nervous one manifested by smarting pains in the situation where the eruption ought to be, but which often precede it for some days, and may be observed to follow the direction of the intercostal nerve. It may happen, however, that these nervous phenomena are only those of inflammation in an occult state.

*Uterine Torticolis.*—As a consequence of partial metro-peritonitis, the uterus sometimes acquires adhesions, which fix its base laterally to the right or the left, and thus give it a strong inclination. Its neck may then be seen twisted to the opposite side in the vagina, in such a manner, that there is a difficulty in discovering the *os tincæ* with the speculum, and from its vicious inclination, it cannot be embraced by the instrument. This condition, which we have observed several times, we denominate uterine, torticolis. If very prominent it constitutes an obstacle to conception.—*Ibid, from Annales de Therapeutique.*

